# Environmental exposures and the risk of multiple sclerosis in Saudi Arabia

**DOI:** 10.1186/s12883-018-1090-8

**Published:** 2018-06-19

**Authors:** Osama Al Wutayd, Ashri Gad Mohamed, Jameelah Saeedi, Hessa Al Otaibi, Mohammed Al Jumah

**Affiliations:** 1 Unaizah College of Medicine, Qassim University, Qassim, Saudi Arabia; 2 Unaizah College of Medicine and Medical Sciences – Qassim University, Unaizah, Qassim Saudi Arabia; 3 King Khalid University Hospital, Riyadh, Saudi Arabia; 4 Princess Norah Bint Abdulrahman University, Riyadh, Saudi Arabia; 5 King Fahad General Hospital, Ministry of Health, Jedda, Saudi Arabia; 6 King Fahad Medical City, MOH, KAIMRC/KSAU-HS, Riyadh, Saudi Arabia

**Keywords:** Multiple sclerosis, Case-control studies, Environmental risk factors, Sun exposure, Fast food, Coffee

## Abstract

**Background:**

Multiple sclerosis (MS) is the most common non-traumatic condition that leads to disability among young individuals. It is associated with demyelination, inflammation, and neurodegeneration within the central nervous system. Information on risk factors of multiple sclerosis is crucial for the prevention and control of the disease. The aim of this study was to determine risk factors of MS among adults in Saudi Arabia.

**Methods:**

A matched multicenter case-control study, including 307 MS patients and 307 healthy controls, was conducted in MS clinics and wards in 3 main cities of Saudi Arabia. Age, gender, and hospital were matched. Information on demographics, family history of MS, past medical and family history, sun exposure at different age periods, tobacco use, diet, consanguinity, and coffee consumption was obtained from self-administered questionnaires. ORs and 95% confidence intervals (CIs) were calculated. A conditional logistic regression model was used to control for potential confounding factors.

**Results:**

The conditional logistic regression adjusted for age and gender showed that being the first child in the family (Adjusted Odds Ratio (AOR) 1.68, 95% CI: 1.03–2.74), having a family history of MS (AOR 5.83, 95% CI: 2.83–12), eating fast food ≥5 times weekly (AOR 2.05, 95% CI: 1.03–4.08), and having had measles (AOR 3.77, 95% CI: 2.05–6.96), were independently associated with an increased risk of MS.

In contrast, eating ≥5 servings of fruit per week (AOR 0.25, 95% CI: 0.16–0.38), drinking coffee daily (AOR 0.46, 95% CI: 0.31–0.68), and having a high level of sun exposure at the primary school level and university level (AOR 0.57, 95% CI: 0.38–0.85 and AOR 0.48, 95% CI: 0.30–0.76, respectively) were independently associated with a decreased risk of MS.

**Conclusions:**

Our study suggested that high levels of sun exposure during primary school and university, consumption of fruits and drinking coffee protect against MS. In contrast, eating fast food was associated with an increased risk of the disease. Encouraging outdoor activity and healthy diets in school, especially for females, is highly recommended.

**Electronic supplementary material:**

The online version of this article (10.1186/s12883-018-1090-8) contains supplementary material, which is available to authorized users.

## Background

Multiple sclerosis (MS) is the most common non-traumatic condition that leads to disability among young individuals, and is associated with demyelination, inflammation, and neurodegeneration within the central nervous system [1]. It is commonly diagnosed between 18 and 40 years of age [2]. MS leads to sensory, motor, and cognitive dysfunctions, which may be progressive, temporary, or permanent. Therefore, multiple sclerosis may have adverse effects on relationships, employment, general well-being and the overall quality of life [3]. The prevalence of MS in the Middle East ranges from low to high. These ranges are dependent on the study setting and the particular population under study [4]. Previously, the Gulf region was thought to have a low prevalence of MS, but recent reports have revealed an increase in the disease prevalence and rate of incidence. However, there are still no registries for MS in the Gulf region [5]. In Saudi Arabia, the studies that are available are mostly hospital-based and there is still no regional or national study that has investigated the prevalence of MS, although some investigators have estimated the prevalence at 30 cases of MS per 100,000 individuals in 2009 [6]. It has been estimated that the prevalence in Saudi Arabia was 40/100,000 in 2008 [7]. The etiology of multiple sclerosis is not yet fully understood, but a single causative event is unlikely. Converging evidence shows that MS is normally the result of autoimmune reactions in genetically susceptible persons following exposure to specific environmental factors [8]. The prevalence of MS is high in the Arabian Gulf region and Saudi Arabia. The risk factors that may be associated with MS in Saudi Arabia remain unclear, while the prevalence of MS is increasing. Thus, this study will provide more knowledge about the determinants of MS in Saudi Arabia.

## Method**s**

A total of 307 MS patients and 307 healthy controls matched for gender and age (± 3 years) were selected from 3 main cities of Saudi Arabia, namely, Riyadh (King Fahad Medical City, King Saud Medical City, and King Fahad National Guard Hospital), Jeddah (King Fahad General Hospital), and Dammam (Dammam Central Hospital). MS patients were recruited from neurological clinics and hospital wards. Controls were healthy companions of patients from medical, surgical, and pediatric clinics and wards. The inclusion criteria for MS patients were those who had clinically definite MS that was diagnosed not more than 4 years from first symptoms and who attended MS clinics or were admitted to the hospital, were aged ≥18 years old, and were able to understand the questionnaire and give informed consent. Exclusion criteria for MS patients were those with clinically definite MS that was diagnosed more than 4 years from symptom onset, aged < 18 years old, and patients with current or concomitant illness that would interfere with the individual’s ability to complete the study (e.g., cognitive impairment). Exclusion criteria for controls were history of MS, aged < 18 years old, and unable to understand the questionnaire and give informed consent. We adopted a structured questionnaire for this study. It included several sections that addressed demographics, family history of MS, sun exposure, current diet, history of breastfeeding, coffee consumption, medical history and family history, tobacco use, obesity, parental consanguinity, and age of onset of menstruation in the case of female patients. With respect to sun exposure, we used the frequency of participants’ outdoor activities at different ages. To ensure the questionnaire’s validity, a pilot study was conducted with 30 patients at the King Khalid University Hospital. This study was used to validate the logistics of data collection, and the clarity of the data collection tool and to estimate the time required to collect the data. The questionnaire took 6 to 12 min to complete. Some questions were modified according to the results of the pilot study. To assure the questionnaire’s content validity, epidemiologists and Consultant Neurologists reviewed it and it was corrected according to their recommendations. The questionnaire was pretested on 30 participants to assess reliability through tests retests on different questionnaire items and Spearman correlations ranged from 0.62 to 1. These pretested groups were not included in the study. Then, data were collected from participants using the self- administered questionnaire. Trained data collectors were responsible for distributing the questionnaire to the participants and collecting them once finished. They were also responsible for the questionnaires’ completeness. Participants filled out the questionnaire but, were supervised closely to address any inquiries they might have and assess whether they left any questions unanswered. The data collectors helped any participants who were unable to fill out the questionnaires by themselves.

SPSS V.21 was used for data entry, management and analysis. The frequencies and percentage of all variables were calculated. The Chi-squared test, Fisher’s exact test, and Student’s t-test were used to test the association between categorical and continuous variables, respectively. The odds ratio associated with each potential risk factor and its 95% confidence interval (CI) was also calculated. To control for confounding variables, conditional logistic regression analysis adjusted for age and gender was applied in various models using MS vs. controls as the dependent variable and other variables as the independent ones. All variables associated with MS status with a *p* value < 0.10 were entered into the model. A p value ≤0.05 was considered statistically significant.

## Results

Out of the 336 MS patients requested to participate, 307 (91%) agreed, and of 340 matched controls, 307 (90%) agreed, resulting in response rates of 91 and 90% for MS patients and controls, respectively. These rates are similar, are considered satisfactory rates, and not statistically significant. Table 1 shows that the majority of subjects (230; 75%) were females, and (77, 25%) were males. The mean age was 32.91 ± 8.82 years for MS patients and 32.89 ± 8.64 years for controls. Forty-two percent of subjects were 18–29 years old. There was no statistically significant difference between MS patients and controls with respect to their level of education (*P* = 0.30). Further, the primary work status over the past 12 months did not significantly differ between patients and controls (*P* = 0.13). The same was true for average household earnings (*P* = 0.19). Among MS patients with a family history of MS, the median difference in age of diagnosis compared to the age of their relatives with MS is 5 years.

**Table 1:** Baseline characteristics of MS patients and their healthy controls

Baseline characteristics	MS patients (*n* = 307)	Controls (*n* = 307)	*P* value
	*n* (%)	*n* (%)	
Female	230 (75)	230 (75)	1
Male	77 (25)	77 (25)	
Age, mean (SD)	32.91 (±8.82)	32.89 (±8.64)	
18–29	128 (41.7)	128 (42)	0.94
30–39	106 (35)	111 (36)	
40–49	56 (18)	50 (16)	
50–59	16 (5)	16 (5)	
60–69	1 (0.3)	2 (1)	
Level of education			
Less than university	142 (46)	158 (52)	0.30
University	149 (49)	130 (42)	
Postgraduate	16 (5)	19 (6)	
Main work status over the last 12 months			
Employed	158 (52)	186 (61)	0.127
Unemployed	23 (8)	15 (5)	
Student	37 (12)	32 (10)	
Housewife	89 (29)	74 (24)	
Average earnings of the household^a^			
< 7500	87 (33)	87 (31)	0.19
7500–12,499	101 (38)	114 (40)	
≥ 12,500	80 (30)	83 (29)	

^a^62 total missing. (most of them did not know), 39 MS patients and 23 controls

Univariate analyses showed that the risk of having MS among first-born children was increased compared to the ≥2nd child in the family (*P* = 0.001), and MS patients were more likely to have a family history of MS (*P* <  0.001). The risk of having MS among those with high sun exposure was decreased compared to those with low sun exposure during primary, intermediate, and secondary schools and university (*P* <  0.001).

We found that MS patients consumed less fruit (*P* <  0.001) and vegetables (*P* = 0.001), more fast food (*P* <  0.001) and dairy products (*P* = 0.03), and less coffee (*P* = 0.001) than controls. MS patients were more likely to have a history of measles (*P* <  0.001) and fewer incidences of thyroid disorder (*P* = 0.04), as well as less parental consanguinity with a first cousin (*P* = 0.03) compared to controls (Table 2).

**Table 2:** Bivariate analysis of variables associated with risk of Multiple Sclerosis

Environmental exposures	Exposed cases/Exposed controls (*n* = 307/307)	Crude OR	95% CI	*P* value
First child in family, yes/no	76/264	2.02	1.34–3.06	0.001
Family history of MS, yes/no	57/294	5.16	2.76–9.64	< 0.001
Sun exposure during primary school, high/low	139/122	0.55	0.40–0.75	< 0.001
Sun exposure during intermediate school, high/low	94/169	0.54	0.39–0.75	< 0.001
Sun exposure during secondary school, high/low	65/192	0.45	0.31–0.64	< 0.001
Sun exposure during university school, high/low^a^	54/184	0.33	0.23 – 0.49	< 0.001
Breastfeeding, yes/no^b^	203/37			
≥ 5 Servings of fruits / week, yes/no	77/145	0.30	0.21–0.42	< 0.001
≥ 5 Servings of vegetables / week, yes/no	145/120	0.57	0.42–0.79	0.001
≥ 5 dates times / week, yes/no	156/146	0.94	0.68–1.29	0.69
≥ 5 red meat times / week, yes/no	38/268	0.97	0.60–1.57	0.90
≥ 5 milk times / week, yes/no	84/239	1.32	0.92–1.91	0.14
≥ 5 Fast food times/week, yes/no	46/291	3.21	1.77–5.80	< 0.001
≥ 5 Dairy products times / week, yes/no	162/172	1.42	1.04–1.96	0.03
Daily coffee intake, yes/no	112/147	0.53	0.38–0.73	< 0.001
Illness or surgical interventions:				
History of measles infection, yes/no	63/283	3.05	1.85–5.02	< 0.001
History of chicken pox infection, yes/no	167/132	0.90	0.65–1.24	0.57
Appendectomy, yes/no	29/268	0.72	0.43–1.19	0.25
Tonsillectomy, yes/no	52/264	1.25	0.81–1.94	0.37
Medical history:				
Type I diabetes mellitus, yes/no	15/285	0.67	0.34–1.31	0.235
Migraine, yes/no	35/269	0.91	0.56–1.49	0.71
Systematic lupus erythematosus, yes/no	1/302	0.19	0.02–1.69	0.22
Rheumatoid arthritis, yes/no	17/289	0.94	0.48–1.86	0.86
Thyroid disorder, yes/no	19/274	0.55	0.30–0.99	0.04
Crohn’s disease, yes/no	1/306	1	0.06–16.06	1
Ulcerative colitis, yes/no	18/294	1.41	0.68–2.93	0.36
Psoriasis, yes/no	7/303	1.77	0.51–6.10	0.361
Family history of medical conditions:				
Type I diabetes mellitus, yes/no	154/154	1.01	0.74–1.39	0.94
Migraine, yes/no	53/249	0.90	0.59–1.35	0.60
Systematic lupus erythematosus, yes/no	10/291	0.61	0.27–1.37	0.23
Rheumatoid arthritis, yes/no	69/239	1.02	0.70–1.49	0.92
≥ 13-year age of menarche, yes/no	98/138	1.11	0.77–1.61	0.57
Tobacco use:				
Current tobacco use, cases/controls	58/52	1.16	0.77–1.76	0.47
Ex-smoker, cases/controls	19/15	1.32	0.84–1.56	0.43
Never smoker, cases/controls	230/240	Ref.		
Passive smoking in childhood, yes/no	113/194	1	0.72–1.39	1
BMI:				
≥ 30, cases/controls	76/74	0.92	0.62–1.37	0.67
25–29.99, cases/controls	90/107	0.75	0.52–1.09	0.13
≤ 24.99, cases/controls	141/126	Ref.		
Parental consanguinity (first cousin), yes/no	63/222	0.67	0.46–0.98	0.03

^a^Not applicable (5 MS cases and 3 controls)

^b^Not know (58 cases and 52 controls)

The following exposures were not found to be significantly associated with MS. These included breastfeeding, age of menstruation, and BMI. With respect to diet, the following were not significant: consumption of dates, red meat, and milk. With respect to family or medical history, the following factors showed no significant association with MS: chicken pox, appendectomy, tonsillectomy, medical history of type I diabetes mellitus, migraine, systematic lupus erythematosus, rheumatoid arthritis, Crohn’s disease, ulcerative colitis, psoriasis, and family history of type I diabetes mellitus, migraine, systematic lupus erythematosus, and rheumatoid arthritis. Tobacco use (cigarette and water pipe) and passive smoking during childhood were not associated with MS (Table S1 shows this in more detail [see Additional file 1]).

A total of 604 participants were included in the conditional logistic regression adjusted for age and gender (Table 3). The rest (10) had incomplete data and were excluded from the model.

**Table 3:** Conditional logistic regression model of environmental exposures with multiple sclerosis risk

Environmental exposures	AOR^a^	95% CI	*P* value
First child in family	1.68	1.03–2.74	0.038
Family history of MS	5.83	2.83–12	< 0.001
≥ 5 Fast food times/week, yes/no	2.05	1.03–4.08	0.042
History of measles infection, yes/no	3.77	2.05–6.96	< 0.001
≥ 5 Servings of fruits / week, yes/no	0.25	0.16–0.38	< 0.001
Daily coffee intake, yes/no	0.46	0.31–0.68	< 0.001
Sun exposure during primary school, high/low	0.57	0.38–0.85	0.006
Sun exposure during university school, high/low	0.48	0.30–0.76	0.002

^a^*AOR* Adjusted odds ratio, included variables in the model are age and gender

This showed that being the first child in the family (Adjusted OR 1.68, 95% CI: 1.03–2.74), having a family history of MS (AOR 5.83, 95% CI: 2.83–12), eating fast food ≥5 times weekly (AOR 2.05, 95% CI: 1.03–4.08), and having had measles (AOR 3.77, 95% CI: 2.05–6.96) were independently associated with an increased risk of MS.

Eating ≥5 servings of fruit per week (AOR 0.25, 95% CI: 0.16–0.38), drinking coffee (AOR 0.46, 95% CI: 0.31–0.68), and having a high level of sun exposure at the primary school level (AOR 0.57, 95% CI: 0.38–0.85) and university (AOR 0.48, 95% CI: 0.30–0.76) were independently associated with a decreased risk of MS.

## Discussion

Birth order is believed to influence the risk of MS. In our study, the risk of MS among first children in the family was increased 1.7-fold compared to those born 2nd or later. We also compared second- and third-born children to first-born children as a reference category and found a protective effect for those born later. Siblings born late in the birth order are exposed to infection earlier than those born earlier and experience greater challenges to their immune systems. The hygiene hypothesis, which states that exposure to infection early in life provides protection from infection, could mean that younger siblings are at decreased risk for developing MS because they have early contact with microorganisms via older siblings. This is consistent with a study that showed that high levels of exposure to infections in younger infant siblings protected against MS [9]. However, other studies have found inconsistent results [10, 11].

The MS risk was 5.8 times greater when there was family history of MS, and this finding was statistically significant, indicating a strong aggregation in MS cases. We calculated the time difference between age at diagnosis for MS patients and their relatives with MS to establish whether this was attributable to shared exposure to environmental factors or to the genes that are responsible for familial aggregation of MS. We believe both genetic and environmental factors play a role in the development of MS. This is unlikely to be due to chance or recall bias, as the clinical features of MS are obvious, and patients and controls would remember them well. This finding is consistent with many studies of MS, such as one conducted in Saudi Arabia that reported that approximately 20% of MS patients have a family history of MS [12]. Two additional studies conducted in Kuwait reported that a larger number of patients had a family history of MS compared to controls [11, 13]. However, in this study, there was no significant difference between first-, second-, and third-degree relatives.

With respect to the frequency of eating fast food, MS patients ate fast food 2 times more often than healthy controls. This result is consistent with a study of MS patients in which sodium consumption was estimated from sodium excretion in urine samples and showed that increased sodium consumption is associated with increased clinical and radiological activity in MS patients [14]. A diet high in salt (sodium chloride) has been found to increase the stimulation of the Th17 lymphocyte both in human and animal models. The Th17 cells produced by a high salt diet seem to be extremely pathogenic and connected to pro-inflammatory cytokines [15]. However, our study is inconsistent with a recent prospective study (NHS) that showed no association between high dietary sodium intake and the risk of MS [16].

Studies in the literature have been inconclusive with respect to the association between measles and MS. A study prospectively collected serum samples from MS patients and revealed no association between MS and measles [17]. Our study revealed that a history of measles in MS patients was 3.8-folds more frequent than in healthy controls. This study is consistent with a matched case-control study of serology among German children with MS [18]. In addition, a study reported that MS patients had measles at an older age compared to controls [19]. A possible explanation for this is delayed exposure to common infectious agents. Thus, infection early in life may protect against MS, while conversely, later infections when the immune system has matured may increase the risk. This is consistent with the inverse relationship between MS risk and birth order. However, in this study, the age of getting the measles infection was not determined, and we suggest the need for further study to assess the association between measles and MS taking into consideration the age of infection.

Fruits and vegetables are rich in numerous vitamins and minerals. Vitamins, in particular, contain antioxidants that have been suggested to play a protective role in the prevention of MS [20]. A case-control study conducted in Serbia showed that the frequency of consumption of fruits and vegetables was increased in healthy individuals compared to MS patients [21]. In this study, healthy controls consumed more fruits and vegetables than MS patients, and the association was found to be significant in a bivariate analysis. Although the frequency of consumption of vegetables in healthy controls was greater than that in MS patients, this association was not significant in the conditional logistic regression. This finding was similar to a case-control study among the Iranian population that reported that consumption of fruit has a protective role against MS, but vegetable consumption was insignificant in a multivariate analysis [22].

In this study, coffee intake among healthy controls was increased compared to MS patients. In the conditional logistic regression, coffee consumption had a protective effect among healthy controls compared to MS patients and this is consistent with a recently published case-control study in Sweden and the U.S. that reported that those who drank four cups of coffee or more each day demonstrated a protective effect [23]. However, one prospective study (NHS) found no association between coffee intake and MS risk and this may be because it was conducted only in females [24]. Several mechanisms have proposed that caffeine appears to decrease the generation of pro-inflammatory cytokines, and has neuroprotective qualities, credited to phosphodiesterase inhibition and non- specific antagonism of adenosine receptors [25, 26].

The comparison of those with low or high sun exposure during different levels of education was used rather than age groups to increase recall of sun exposure Nevertheless, primary, intermediate, secondary school, and university correspond to the age groups, 7 to 12, 13 to 15, 16 to 18, and 19 to 22 years, respectively. Sun exposure in primary school was associated with reduced risk of MS. This finding is consistent with several studies, such as one conducted in Tasmania that found that increased sun exposure during childhood and early adolescence was associated with a decreased risk of MS [27]. Another study that found an association between sun exposure in childhood and the risk of monozygotic twins with MS reported similar results [28]. Moreover, sun exposure during university was associated with a reduced risk of MS. The mechanism of sun exposure can be explained by Vitamin D, which has been proposed to be the main mediator of this protective effect [29]. Vitamin D receptors bind thousands of genomic sites in immune systems in lymphoblastoid cells, and there is a direct correlation between vitamin D levels and vitamin D receptor binding sites, which bind to fewer sites in primary CD4+ T cells in individuals with low vitamin D levels compared to individuals with normal vitamin D levels [30, 31]. Nevertheless, a population-based case-control study endorses the theory that UVR exposure contributes to reducing MS risk independently of its consequences on vitamin D levels [32]. Moreover, research in Australia recommended that both solar emission and vitamin D have individual protective effects on MS development [33]. There are several pathways through which UVR may influence immune reactions that are independent of vitamin D production, for example, UVB contact influences universal immune responses and assuages systemic autoimmunity through the introduction of regulatory T cells and skin- derived tolerogenic dendritic cells [34].

In this study, current smokers were found more often in the MS cases than in healthy controls; however, this was not statistically significant. This may be due to fact that the majority of MS patients in our study were women and that smoking is more common among men than women in our community. A recent meta-analysis including 10 studies showed that cigarette smoking appeared to increase the risk of MS to a greater extent in men than in women. Additionally, the majority of cigarette smokers were not heavy smokers. We consider that this may be related to MS patients quitting smoking after being diagnosed with the disease. However, this may be unlikely especially in cases of ex-smokers, where differences were not statistically significant between MS patients and controls. On the other hand, tobacco use in our region may have little influence on the development of MS, and this finding is consistent with two studies reported from Kuwait [11, 35].

In our study, as in all case-control studies, there are several limitations. Case control studies are subject to recall bias which is a potential threat to the validity of the results. In the present study, we recruit only MS cases who were within 4 years of diagnosis to minimize the risk for misclassified answers.

Information was obtained retrospectively, with some of the exposures referring to years long before the study, and thus, this study may have exposure misclassification. However, for example, we used school level rather than age period to increase the recall of MS patients and controls regarding sun exposure. Additionally, we asked about viral infections with obvious clinical features, such as measles and chicken pox, which participants could recall easily. Other epidemiological studies need to be carried out to know others that were not assessed in the present study such as Epstein-Barr virus, since this is a major risk factor in other populations. Diet assessment is one limitation in this study, since participants could have changed their current diet since diagnosis. Selection bias is a threat if controls are not representative of the population from which the cases arose. In this particular study, controls were specifically selected from the same hospital as the MS cases. Additionally, selection bias could be considered since controls may have MS as there is a relatively long latency period. However, this is unlikely since MS is not common in the general population. The matched analysis helped to control for these design characteristics.

## Conclusions

Our study suggested that high levels of sun exposure during primary school and university, consumption of fruits and drinking coffee protect against MS. In contrast, eating fast food was associated with increased risk of the disease. Encouraging outdoor activity and healthy diets in school, especially for females, is highly recommended.

## Additional file

Additional file 1: **Table S1.** Bivariate analysis of variables associated with risk of Multiple Sclerosis. (PDF 338 kb)

## Abbreviations

AOR: Adjusted odds ratio
CI: Confidence interval
MS: Multiple sclerosis
NHS: Nurses’ health study
ORs: Odd ratios
Th17: T helper type 17 lymphocyte
US: United States
UVB: Ultraviolet B
UVR: Ultraviolet radiation
