# Supplementary material for: Environmental exposures and the risk of multiple sclerosis in Saudi Arabia
Source: BMC Neurol. 2018 Jun 19;18:86. doi: 10.1186/s12883-018-1090-8 (PMC6006694; doi:10.1186/s12883-018-1090-8)
Supplement: Supplementary file 1 — Table S1. Bivariate analysis of variables associated with risk of Multiple Sclerosis. (PDF 338 kb) [file 12883_2018_1090_MOESM1_ESM.pdf]

Additional file 1. Table S. Bivariate analysis of variables associated with risk of Multiple Sclerosis.

		MS N=307 (%)	Control N=307 (%)	OR	95% CI	P value
First child in family	Yes	76 (25)	43 (14)	2.02	1.34 – 3.06	0.001
	No	231 (75)	264 (86)			
Family History of MS	Yes	57 (19)	13 (4)	5.16	2.76 – 9.64	<0.001
	No	250 (81)	294 (96)			
Sun exposure during primary school	High	139 (45)	185 (60)	0.55	0.40 - 0.75	<0.001
	Low	168 (55)	122 (40)			
Sun exposure during intermediate school	High	94 (31)	138 (45)	0.54	0.39 - 0.75	<0.001
	Low	213 (69)	169 (55)			
Sun exposure during secondary school	High	65 (21)	115 (38)	0.45	0.31 - 0.64	<0.001
	Low	242 (79)	192 (63)			
Sun exposure during university*	High	54 (18)	120 (40)	0.33	0.23 - 0.49	<0.001
	Low	248 (82)	184 (60)			
Servings of Fruits / week	≥ 5	77 (25)	162 (53)	0.30	0.21 - 0.42	< 0.001
	< 5	230 (75)	145 (47)			
Servings of vegetables / week	≥ 5	145 (47)	187 (61)	0.57	0.42 - 0.79	0.001
	< 5	162 (53)	120 (39)			
Dates Times / week	≥ 5	156 (51)	161 (52)	0.94	0.68 - 1.29	0.686
	< 5	151 (49)	146 (48)			
Red meat Times / week	≥ 5	38 (12)	39 (13)	0.97	0.60 - 1.57	0.903
	< 5	269 (88)	268 (87)			
Fast food Times / week	≥ 5	46 (15)	16 (5)	3.21	1.77 – 5.80	< 0.001
	< 5	261 (85)	291 (95)			
Milk Times / week	≥ 5	84 (27)	68 (22)	1.32	0.92 – 1.91	0.135
	< 5	223 (73)	239 (78)			
Dairy products Times / week	≥ 5	162 (53)	135 (44)	1.42	1.04 – 1.96	0.036
	< 5	145 (47)	172 (56)			
Milk type**	Breast milk	203 (76)	218 (82)	0.75	0.47 – 1.2	0.230
	Artificial formula	46 (17)	37 (41)			
Coffee intake	yes	112 (36)	160 (52)	0.53	0.38 – 0.73	<0.001
	no	195 (64)	147 (48)			
Measles	Yes	63 (21)	24 (8)	3.05	1.85 - 5.02	<0.001
	No	244 (79)	283 (92)			
Chicken pox	Yes	167 (54)	175 (57)	0.90	0.65 - 1.24	0.57
	No	140 (46)	132 (43)			
Appendectomy	Yes	29 (9)	39 (13)	0.72	0.43 – 1.19	0.25
	No	278 (91)	268 (87)			
Tonsillectomy	Yes	52 (17)	43 (14)	1.25	0.81 – 1.94	0.37
	No	255 (83)	264 (86)			

**Medical history of**						
Type I diabetes mellitus	Yes	15 (5)	22 (7)	0.67	0.34 – 1.31	0.235
	No	292 (95)	285 (93)			
Migraine	Yes	35 (11)	38 (12)	0.91	0.56 – 1.49	0.708
	No	272 (89)	269 (88)			
Systematic lupus erythematosus	Yes	1 (0.3)	5 (2)	0.19	0.02 – 1.69	0.22
	No	306 (99.6)	302 (98)			
Rheumatoid arthritis	Yes	17 (6)	18 (6)	0.94	0.48 – 1.86	0.86
	No	290 (94)	289 (94)			
Thyroid disorder	Yes	19 (6)	33 (11)	0.55	0.30 – 0.99	0.042
	No	288 (94)	274 (89)			
Crohn's disease	Yes	1 (0.3)	1 (0.3)	1	0.06 – 16.06	1.00
	No	306 (99.6)	306 (99.6)			
Ulcerative colitis	Yes	18 (6)	13 (4)	1.41	0.68 – 2.93	0.357
	No	289 (94)	294 (96)			
Psoriasis	Yes	7 (2)	4 (1)	1.77	0.51 – 6.10	0.361
	No	300 (98)	303 (99)			
**Family history of**						
Type I diabetes mellitus	Yes	154 (50)	153 (50)	1.01	0.74 – 1.39	0.936
	No	153 (50)	154 (50)			
Migraine	Yes	53 (17)	58 (19)	0.90	0.59 – 1.35	0.600
	No	254 (83)	249 (81)			
Systematic lupus erythematosus	Yes	10 (3)	16 (5)	0.61	0.27 – 1.37	0.229
	No	297 (97)	291 (95)			
Rheumatoid arthritis	Yes	69 (22)	68 (22)	1.02	0.70 – 1.49	0.923
	No	238 (78)	239 (78)			
Tobacco use	Current smokers	58 (19)	52 (17)	1.16	0.77 – 1.76	0.47
	Ex-smokers	19 (6)	15 (5)	1.32	0.84 – 1.56	0.43
	Never smokers	230 (75)	240 (78)	Ref.		
Passive smoking in childhood	Yes	113 (37)	113 (37)	1	0.72 – 1.39	1
	No	194 (63)	194 (63)			
BMI	≥ 30	76 (25)	74 (24)	0.92	0.62 – 1.37	0.67
	25 – 29.99	90 (29)	107 (35)	0.75	0.52 – 1.09	0.13
	≤ 24.99	141 (46)	126 (41)	Ref.		
Parental consanguinity (first cousin)	Yes	63 (21)	85 (28)	0.67	0.46 – 0.98	0.03
	No	244 (79)	222 (72)			
Age of menstruation	9-12 Year	98 (43)	92 (40)	1.11	0.77 – 1.61	0.57
	≥ 13 Year	132 (57)	138 (60)			

* Not applicable (5 MS cases and 3 controls).

** Not know (58 MS cases and 52 controls)
